# Prioritization of strategies to approach the judicialization of health in Latin America and the Caribbean

**DOI:** 10.1590/S1518-8787.2016050005728

**Published:** 2016-08-26

**Authors:** Carlos Eduardo Pinzón-Flórez, Evelina Chapman, Leonardo Cubillos, Ludovic Reveiz

**Affiliations:** I Grupo de Investigación en Salud. Universidad de La Sabana. Chía, Colombia; II Organização Pan-americana de Saúde. Brasília, DF, Brasil; III World Bank Institute. Washington, DC, USA; IV Department of Knowledge Management, Bioethics and Research. Pan American Health Organization. Washington, DC, USA

**Keywords:** Judicial Decisions, Jurisprudence, Patient Rights, Right to Health, Health Priorities, legislation & jurisprudence, Health Systems

## Abstract

**OBJECTIVE:**

To describe strategies that contribute to the comprehensive approach to the judicialization of health in countries of Latin America and the Caribbean.

**METHODS:**

A search was structured to identify articles presenting strategies to approach the judicialization of health. A survey was designed, which included actors of the health system and judiciary sector. We prioritized the strategies qualified by more than the 50.0% of the participants as “very relevant”. Strategies were categorized according to: governance, provision of services, human resources, information systems, financing, and medical products.

**RESULTS:**

We included 64 studies, which identified 50 strategies, related to the sub-functions and components of health systems. Of the 165 people who answered the survey, 80.0% were aged 35-64 years. The distribution of men and women was homogeneous. Half of the respondents were from Colombia (20.0%), Uruguay (16.9%), and Argentina (12.7%). We prioritized strategies that addressed aspects of generation of useful scientific evidence for decision making according to the health needs of the population, empowerment for the society, and creating spaces for discussion of measures of inclusion or exclusion of health technologies. The executive and judiciary decision makers prioritized questions that dealt with strategies that would ensure accountability.

**CONCLUSIONS:**

The results of this study contribute to the identification of effective strategies to approach the phenomenon of judicialization of health, guaranteeing the right to health.

## RESUMEN

**OBJETIVO:**

Describir estrategias que contribuyan al abordaje integral de la judicialización de la salud en países de América Latina y El Caribe.

**MÉTODOS:**

Se estructuró una búsqueda para identificar artículos que presentaran estrategias para el abordaje de la judicialización en salud. Se diseñó una encuesta, en donde se incluyeron actores del sistema de salud y del sector judicial. Se priorizaron las estrategias calificadas por más del 50,0% de los participantes como “muy relevantes”. Se categorizaron las estrategias según: gobernanza, prestación de servicios, recursos humanos, sistemas de información, financiación y productos médicos.

**RESULTADOS:**

Se incluyeron 64 estudios en los cuales se identificaron 50 estrategias, relacionadas con las sub-funciones y componentes de los sistemas de salud. De las 165 personas que respondieron la encuesta, el 80,0% tenían entre 35-64 años. La distribución de hombres y mujeres fue homogénea. La mitad de los respondientes fue de Colombia (20,0%), Uruguay (16,9%) y Argentina (12,7%). Se priorizaron mayormente las estrategias que abordaron los aspectos de generación de evidencia científica útil para toma de decisión según las necesidades de salud de la población, el empoderamiento para la sociedad, y la generación de espacios de discusión de las medidas de inclusión o exclusión de tecnologías de salud. Los tomadores de decisión de la rama ejecutiva y judicial priorizaron las preguntas que abordaron las estrategias que garantizaran la rendición de cuentas.

**CONCLUSIONES:**

Los resultados de este estudio contribuyen a la identificación de estrategias efectivas para el abordaje del fenómeno de la judicialización en salud, garantizando el derecho a la salud.

## INTRODUCTION

The right to health is well stablished in most constitutions of the countries of Latin America and the Caribbean, as well as in numerous international and regional human rights treaties. This obliges the States to ensure, among others: timely and appropriate availability of health care; healthy and safe work conditions; adequate housing; and nutritious foods. When the States cannot guarantee this right properly, they must adopt measures in accordance with the principle of progressive realization, i.e., to progress in the most expeditious and efficient way possible using the maximum of the resources available^a^.

The legitimate role of a court in the process of litigation on health is to represent the democratic processes and institutional capacity of the State to exercise them. This evolution demonstrates a process of social maturation of the States, recognizing human rights and their enforceability of protection, and clearly establishing governance and governability in terms of establishing an inclusive mechanism of accountability in the society^12^ ^,^ ^20^ ^,^ ^b^.

In 2005, universal health coverage became the objective of all the health care systems of countries members of the World Health Organization (WHO), as an effective strategy to achieve access to the health services needed to establish the health of populations, without incurring in any impoverishing economic risk by individuals. In the medium and long term, to increase the coverage in health services to financial sustainability will enable the improvement of health and well-being, and promote sustainable human development^c^. Several countries around the world have presented an accelerated demand of the population by universal quality health services. This increase in demand is not often harmonized with the development of systems, and is associated with a deterioration in the provision of health services, either by endogenous or exogenous factors to the system itself^14^ ^c^ ^,^ ^d^ ^,^ ^e^.

Litigations in health have been efficient for acquiring such health services in an effective way. These mechanisms are being increasingly used. This is reflected in the exponential increase in the number of court judgments related to the requirement of fulfilment of the right to health by the State. As a result, the judiciary has participated more in the definition and redefinition of health policies, and has also been evolving and redefining: the type of responses, the functions that must be exercised within the health system, and the role within the exercise of the right to the health^13^ ^,^ ^39^. While they may be a medium term solution to guarantee the individual right and access to health, judicial tutelage and protections, criminal and civil, for example, may require the increase of public financing of health in a sustainable and effective way, to ensure that it does not impacts negatively the financial sustainability of the health system^13^ ^,^ ^39^.

The aim of this article was to describe strategies that contribute to the comprehensive approach to the judicialization of health in countries of Latin America and the Caribbean.

## METHODS

A study with two methodological approaches was carried out. The first was a review of the literature to identify strategies that addressed the judicialization of health. The second was a survey to prioritize the identified strategies.

### Literature Search

An advanced search of the literature (January 2000 – July 2013) was performed in the following databases: PubMed; Cochrane Library, and Lilacs; as well as Google Scholar. As there are no validated search strategies for this topic, the following keywords and synonyms, using the PubMed search engine were identified: PubMed: ((“litigation”[tw] OR judicialization [tw] OR lawsuits [tw] OR judiciary [tw] OR legislation and jurisprudence [Subheading] OR jurisprudence [tw] OR court [tw])) AND ((“health”[MeSH Terms] OR “health”[All Fields])) AND ((Latin america* OR South America* central America* OR carribbean* OR Anguilla OR (Antigua Barbuda) OR Argentina OR Aruba OR Bahamas OR Barbados OR Belize OR Bermuda OR Bolivia OR Brazil OR brasil OR (British Virgin Islands) OR (Cayman Islands) OR Chile OR Colombia OR (Costa Rica) OR Cuba OR Dominica OR (Dominican Republic) OR (republica dominicana) OR “El Salvador” OR Ecuador OR (French Guiana) OR Grenada OR Guadalupe OR Guatemala OR Guyana OR Haiti OR Honduras OR Jamaica OR Martinique OR Mexico OR Montserrat OR (Netherlands Antilles) OR Nicaragua OR Panama OR Paraguay OR Peru OR (Puerto Rico) OR (Saint Kitts Nevis) OR (Saint Lucia) OR (Saint Vincent Grenadines) OR Suriname OR (Trinidad Tobago) OR Uruguay OR Venezuela)).

In addition, the bibliographic references of the identified studies were revised for additional references. One reviewer assessed the titles and abstracts identified by the search, and two reviewers independently assessed the studies that met the inclusion criteria, namely: (a) studies whose theme was the judicialization of health; (b) studies that incorporate mechanisms or strategies, or both, to deal with litigations in different health systems in the region; and (c) evaluation of the implementation of structural sentences of courts. The websites of the Ministries of Health and relevant institutions were also reviewed. The search was limited to the languages Spanish, French, English and Portuguese, and was geographically restricted to Latin America and the Caribbean.

### Development of the Instrument (Survey)

A group formed by experts in epidemiology, public health, health systems and services, and global health composed the identified studies. Based on the findings of the review, we developed a map of the different strategies evaluated or proposed by the authors of the studies (Table 1).

**Table 1:** Strategies by category of analysis.

Question	General measures
Q1	1. Is the generation of spaces for discussion of the actors in the health system, including representatives of patients, health professionals, and vulnerable populations, an effective measure to prevent litigations in health?
Q2	2. Is the funding, by the State, of the creation of scientific and civilians associations to strengthen decision making in health, an effective measure to prevent litigations in health?
Q3	3. Is the creation of a public policy that clarifies explicitly the technical and administrative management of the procedures, inputs, interventions, or benefits not included in the set of benefits of the health system, an effective measure to prevent litigation in health?
Q4	4. Is the creation of a strategy that clarifies explicitly which medical inputs and procedures are excluded of the plan of attention in health, as well as the reason of exclusion, an effective measure to reduce litigations in health?
Q5	5. Are explicit strategies to protect the regulatory public institutions of health (for example, systems of incentives and sanctions) from corruption, effective measures to reduce litigation?
Q6	6. Is the institutionalization and/or strengthening of committees in the governing institution (Ministry or Secretariat of Health), which monitor and audit the activities of buying and selling of health services, as well as appropriate provision of services by health professionals, an effective measure to reduce litigations in health?
Q7	7. Is the creation of the figure and the institutionalization of the advocacy of users or patients, an effective strategy to reduce litigations in health?
Q8	8. Is the creation of spaces such as scientific advisory committees to the courts, with the purpose of supporting legal decisions with scientific evidence, an effective measure for decision-making, and also for the regulation of litigation in health?
Q9	9. Is to grant punitive power to the institutions of surveillance and control of the health system for the compliance of processes and the provision of health services an effective measure to reduce litigations in health?

Leadership and governance	

Q10	1. Is the definition of a public policy of surveillance of litigations in health an useful strategy to regulate judicialization of health?
Q11	2. Is the creation of observatories for knowledge, monitoring, and decision making of litigation in health, integrating actors of the health, executive, and judicial sectors, an effective strategy to regulate the judicialization of health?
Q12	3. Is the training and advice by qualified personnel of the Ministry of Health to the members of courts for decision-making in relation to the right to health, an effective strategy to regulate the judicialization of health?
Q13	4. Is training and advice by qualified staff of the scientific community in health (academic staff, researchers) to the members of the courts, an effective strategy to regulate the judicialization of health?
Q14	5. Can the continuous updating of the services and/or set of features based on the needs and priorities of public health regulate litigations in health?
Q15	6. Is the establishment of priorities in health to prevent possible budgetary expenditure and, therefore, catastrophic expenditure in high cost disease an effective measure for the regulation of the judicialization of the right to health?
Q16	7. Can the implementation of institutions or agencies for evaluating health technology that have the capacity to regulate the market in health and the entry of new technologies be an effective mechanism for the regulation of judicialization of health?
Q17	8. Can the reduction of the asymmetry of information between the offer and demand of health care (access, quality, and satisfaction) be a strategy to ensure protection to the consumer and, accordingly, to regulate the judicialization of health?
Q18	9. Can the establishment of a responsible mechanism of conflict resolution in health be an effective strategy for the regulation of the judicialization of health?
Q19	10. Are to properly inform patients or family members on the right to health, the duties of the State and the system of health in relation to the guarantee of this right, strategies of empowerment for the society that will ensure equity and therefore regulate of litigations in health?
Q20	11. Is the planning and implementation of a monitoring and control system based on the guarantee of coverage of health insurers and health care providers a fair, effective, and efficient measure to reduce litigations in health?
Q21	12. Is the development of a strategic plan for the distribution and supplying of pharmacies in the nation and regional levels an effective and efficient measure to reduce litigation in health?

Provision of services	

Q22	1. Is the explicit guarantee of financing and access to health services an action that can reduce the probability of judicialization of health?
Q23	2. Is implementing strategies of primary health care an effective and efficient mechanism to grant equity in the access and financing of health and, consequently, can it be a measure to reduce litigation in health?
Q24	3. Is the implementation of strategies to increase access to health care in rural areas (eHealth, first level health centers in rural areas or with difficult access, economic incentives for the retention of health professionals in rural areas, transportation in areas with geographic access barriers) an effective measure to regulate litigations in health?
Q25	4. Is the redistribution of financial resources for the care of vulnerable populations a measure that improves equity in health and, likewise, would reduce litigation in health?
Q26	5. Is the approval of the plan of services between different sub-sectors in segmented health systems an effective strategy to reduce inequality in access and, therefore, regulate litigations in health?
Q28	7. Is optimizing the waiting times for the provision of health services an effective measure to reduce the number of litigations in health?
Q29	8. Is the hiring of additional health personnel to optimize health care an effective measure for the reduction of litigation in health?
Q30	9. Are the mechanisms of hiring of health services by legal mechanisms effective strategies for the reduction of litigation in health?
Q31	10. Is the generation and implementation of a system of incentives for health care quality and efficiency by health professionals an effective measure to reduce litigation in health?
Q32	11. Is the establishment of inter-agency agreements for the provision of health services an effective measure to ensure the efficient access to such services and, consequently, to reduce litigation in health?
Q33	12. Is the implementation of a system of incentives to offer health services, by insurers or providers, a strategy that ensures the access to services and, therefore, guarantee the right to health effectively and efficiently?
Q34	13. Is encouraging the prescription of generic drugs an effective measure for the regulation of litigations in health?

Human resources	

Q35	1. Is having undergraduate and graduation programs in Law Schools focused on health a good strategy to regulate litigation in health?
Q36	2. Is the timely training of health professionals and judges for decision-making in cases of judicial tutelage an effective strategy to regulate litigation in health?
Q37	3. Is the training of professionals in audit processes and health management an effective strategy to control the quality and management of health services delivery and, likewise, to prevent litigations in health?
Q38	4. Is the establishment of a certification system of professionals and of accreditation of the institutions of health for the provision of quality health services and an effective strategy to regulate litigations in health?
Q39	5. Can developing a strategic plan for the training and distribution of human resources in your country be an effective strategy to reduce and regulate litigation in health?

Information	

Q40	1. Can the development and implementation of clinical practice guidelines or protocols be a strategy to regulate litigations in health?
Q41	2. Is the development of a national plan for the development of guides of clinical practice, protocols of management or guidelines for the provision of health services an effective strategy to regulate litigations in health?
Q42	3. Can the implementation of an information system for decision-making on access, training, and distribution of human resources in health, drug supply and acquisition of new technologies, be an effective strategy to regulate ltigations in health?
Q43	4. Is the implementation of a national system of pharmaceutical information on the access, use, quality and prices of medicines an effective strategy to regulate litigations in health?
Q44	5. Will the creation and management of an information system on the juridical processes in health allow the population to know and characterize these processes, as well as helping decision-making of the actors involved in them?
Q45	6. Is the generation of scientific evidence from research on the impact of judicialization in the management of the health system and the judicial system an effective strategy to regulate litigations in health?
Q46	7. Is the generation of scientific evidence oriented to the identification of health needs of populations an effective measure for the strategical planning of health systems and, therefore, for the regulation of litigations in health?

Financing	

Q47	1. Is the calculation of the additional cost of the introduction of new interventions or technology in the health system an efficient and effective strategy to regulate litigations in health?
Q48	2. Can the creation of a subsystem of financing for high-cost diseases, or that may incur in budgetary or catastrophic expenditure be an effective strategy to regulate litigations in health?
Q49	3. Can the establishment of a threshold of willingness to pay for access to a new health technology be an effective and efficient strategy to regulate litigations in health?
Q50	4. Is strengthening the aid from multinational agencies for the purchase of inputs or supplies of health services an effective measure to regulate litigations in health?

#### Categorization of Strategies

The strategies were classified in relation to the functions of the health system, from the perspective of thinking proposed by WHO^e^: 1) governance, 2) funding, 3) access to medical products and technology, 4) information systems, 5) human resources, and 6) provision of services^21^. The instrument was developed in the SurveyMonkey^®^ program. The strategies were drafted and revised until a consensus was obtained among all members of the group, ensuring that they were short and specific, not ambiguous and established consistent and measurable strategies, thus developing the final instrument. The strategies were written in Spanish and English.

#### Population and Participants

The countries of Latin America and the Caribbean were selected, and in each country decision makers of all three branches of public power (legislative, judicial, and executive) were selected. The participants were identified by observation of the region’s health systems of health, including the regional initiative on prioritization, equity and constitutional mandates for the region of the Americas, driven by the World Bank Institute^f^, and a search in PubMed and Lilacs of actors key in this field in countries of Latin America and the Caribbean. Seventeen participants belonged to countries that are not part of Latin America and the Caribbean. However, they are decision makers and researchers that have contributed on legislation and knowledge over judicialization of health in the region, identified in the literature search. The characteristics of the participants are summarized in Table 2.

**Table 2:** Characteristics of the participants.

Characteristics	n	%
Sex		
Male	86	52.2
Female	79	47.8
Total	165	100
Age (years)		
18 to 24	1	0.6
25 to 34	25	15.1
35 to 44	40	24.2
45 to 54	52	31.5
255 to 64	38	23.0
> 65	9	5.4
Total	165	100
Job position		
Judiciary sector	21	12.7
Executive sector	8	4.8
Legislative sector	1	0.6
Public health sector	71	43.0
Private health sector	40	24.2
International Organization	24	14.5
Total	165	100
Country of origin		
Argentina	21	12.7
Brazil	17	10.3
Canada	1	0.6
Chile	7	4.2
Colombia	33	20.0
Costa Rica	10	6.06
Ecuador	2	1.2
El Salvador	1	0.6
Spain	5	3.03
United States of America	9	5.4
Guatemala	1	0.6
Honduras	1	0.6
Morocco	2	1.2
Mexico	5	9.0
Nicaragua	1	0.6
Peru	9	5.4
Dominican Republic	1	0.6
Uruguay	28	16.9
Trinidad and Tobago	1	0.6
Total	165	100

#### Statistical Analysis

To assess the validity of the construct of the designed instrument, a factor analysis was made regarding the main components. A descriptive analysis of the responses given by participants was performed, taking into account the current occupation and the country where they work. A bivariate analysis was also performed, for the categorical variables current occupation, country where the participant works and degree of prioritization using the X^2^ test. For all statistical analyses, statistical significance values of p < 0.05 were established. The statistical program Stata 12^®^ was used to perform these tests.

## Ethical Aspects

All participants were contacted virtually and simultaneously by sending them the consent form to participate and access to the survey structured in the SurveyMonkey^®^ program. In the header of the survey the purpose of the prioritization exercise and how results would be used were explained. An e-mail contact and a specific field was available to participants to include questions or formulate their concerns. Two reminders of the survey were sent. The proposal was approved by the Ethics Committee of the Pan-American Health Organization (PAHO-ERC – 2013-05-009).

## RESULTS

### Literature Search

We identified 2,245 references. Of these, 2,014 were excluded after application of the eligibility criteria by title and abstract. We excluded 156 articles for being duplicates, and 11 for not reporting strategies of intervention to the research problem addressed. Finally, we evaluated the full text of 64 articles^1^ ^-^ ^6^ ^,^ ^8^ ^-^ ^10^ ^,^ ^11^ ^-^ ^39^ ^,^ ^4^ ^1^ ^-^ ^46^ ^,^ ^48^ ^-^ ^54^ ^,^ ^a^ ^,^ ^b^ ^,^ ^c^ ^,^ ^f^ ^,^ ^g^ ^,^ ^h^ ^,^ ^i^ ^,^ ^j^ ^,^ ^k^ ^,^ ^l^ ^,^ ^m^ ^,^ ^n^ ^,^ ^o^ ^,^ ^p^ ^,^ ^q^ that identified intervention proposals to address the phenomenon of the judicialization of health, as shown in the Figure. All eligible studies were in Spanish, English and Portuguese. The approach of judicialization of health depends on the conceptualization of the right to health and the characteristics of the health care system. All those studies addressed judicialization as a consequence of the relations and performance of the system of health and its civic duty. The majority of legal processes filed was to ensure access to medication (73.0%), and conduction of healing procedures (22.0%) and public health events (5.0%). All litigations were due to medications or procedures. In Colombia^1^ ^,^ ^4^ ^,^ ^19^ ^,^ ^26^ ^,^ ^29^ ^,^ ^32^ ^,^ ^39^ ^,^ ^49^ ^,^ ^g^ ^,^ ^h^ ^,^ ^i^ ^,^ ^j^ ^,^ ^k^ ^,^ ^l^ ^,^ ^m^ ^,^ ^n^ and Costa Rica^35^ ^,^ ^39^, those medications were not included in the plan of benefits. In Brazil, while they were inside the plan of benefits, there was no supply within the evaluated municipalities^3^ ^,^ ^22^ ^,^ ^24^ ^,^ ^30^. The countries with the largest number of publications were Brazil (n = 32, 50.0%)^3^ ^,^ ^5^ ^-^ ^9^ ^,^ ^12^ ^,^ ^1^ ^7^ ^-^ ^18^ ^,^ ^22^ ^-^ ^25^ ^,^ ^27^ ^,^ ^30^ ^,^ ^31^ ^,^ ^34^ ^,^ ^36^ ^-^ ^39^ ^,^ ^42^ ^,^ ^51^ ^,^ ^53^ ^,^ ^o^ ^,^ ^p^, Colombia (n = 21, 32.8%)^1^ ^,^ ^2^ ^,^ ^19^ ^,^ ^26^ ^,^ ^29^ ^,^ ^32^ ^,^ ^39^ ^,^ ^41^ ^,^ ^49^ ^,^ ^g^ ^,^ ^i^ ^,^ ^j^ ^,^ ^k^ ^,^ ^l^ ^,^ ^m^ ^,^ ^n^, Costa Rica (n = 6, 9.3%)^35^ ^,^ ^39^, Chile (n = 2, 3.1%)^8^ ^,^ ^9^ ^,^ ^54^, and Argentina (n = 3, 4.7%)^10^.

**Figure:**
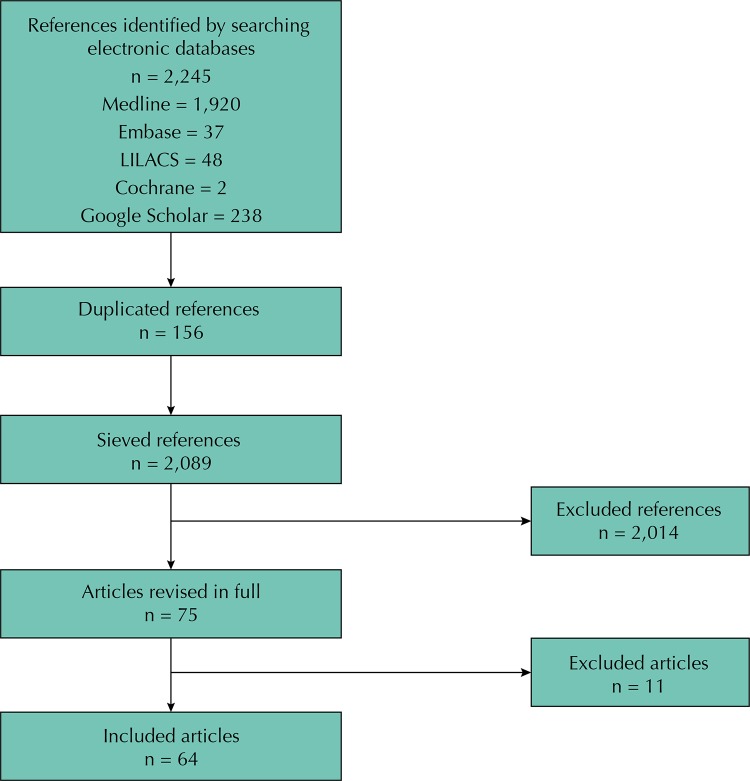
Flowchart of the search strategy.

### Construction of the Collection Instrument

The revision of the articles resulted in 91 proposed strategies, which were evaluated by the group of authors. We excluded 41 of them for: being duplicated; being too general proposals, which prevented implementation; not being applicable in the current context; or presenting ambiguity or inconsistency. Finally, we identified 50 proposals for the strategies, classified as follows: 9 (18.0%) general or systemic measures; 12 (24.0%) governances and leaderships; 13 (26.0%) provision of health services; five (10.0%) human resources for health; seven (14.0%) information; and four (8.0%) financing (Table 3). In the factor analysis by main components with 50 variables and 165 observations, the items were grouped into six factors that explained the variance of the items by 50.0%, according to the proposed theoretical classification. The two first factors presented the greater common variance (15.0%) and explained the construct; this could be due to several proposals of factor 1 “general measures” being related to factor 2 “governance and leadership”. The six main factors with their respective items were: 13 items for factor 1 “general measures” (eigenvalue 12.81); 11 items for factor 2 “governance and leadership” (eigenvalue 2.81); four items for factor 3 “human resources for health” (eigenvalue 2.32); 11 items for the factor 4 “provision of health services” (eigenvalue 1.89); six items for factor 5 “information” (1.75 eigenvalue); and three for the factor 6 “financing” (eigenvalue 1.61), X^2^ = 2452.29 (p < 0.001).

**Table 3:** Analysis according to categories.

Category	Global		Prioritized	
	
	n	%	n	%
General measures	9	18.0	4	40.0
Leadership and Governance	12	24.0	2	20.0
Provision of health services	13	26.0	1	10.0
Human resources of health	5	10.0	1	10.0
Information	7	14.0	2	20.0
Financing	4	8.0	0	0

Total	50	100	10	100

### Prioritization of Strategies according to Categories

The participants prioritized, firstly, the category “general measures”, which involves the establishment of policies and scientific frameworks for the management of the system and intersectoral participation. Secondly, they prioritized the “governance and leadership” category, for the management of health institutions, with emphasis on guaranteeing the right to health and the allocation of priorities in health. A third category prioritized at the same level as the previous one was “information”, which highlights the importance of the use of the evidence for the sets of benefits. Finally, there are the categories “human resources for health” and “provision of health services”, which highlight processes of the quality of care (Table 3).

### Prioritization of Strategies according to Participants

From the 10 prioritized strategies (Table 4), the first three were: (1) to create spaces for discussion for decision-making at different levels of power in the health care system, (2) to duly inform the right to health to patients and families, and (3) to generate scientific evidence focused on the identification of the health needs of the populations. The following two priority strategies are correlated and propose the creation of technical and scientific committees and the training of health professionals and judges, to support the decisions made in the courts, based on scientific evidence, if there is a process of judicialization. Then, the participants prioritized three strategies: the generation of a public policy of regulation of new technologies; continuous updating of the health system’s plans of benefits; and updating of the explicit regulatory mechanisms of evaluation and inclusion of new technologies in the plan of benefits. Finally, priority was given to two strategies related to the optimization of the action of the health professionals in the provision of services: the development and implementation of clinical practice guidelines to standardize health interventions; and the optimization of times of care by health professionals in required services.

**Table 4:** General ranking of strategies prioritized as “very important”.

Ranking	Judicialization of health	Proposed strategy	Response rate	Percentage

	Category			
P1	General measures	Is the generation of spaces for discussion of the actors in the health system, including representatives of patients, health professionals, and vulnerable populations, an effective measure for the prevention of litigations in health?	82/127	64.5
P2	Governance	Are to properly inform patients or family members on the right to health, the duties of the State and the system of health in relation to the guarantee of this right, strategies of empowerment for the society that will ensure equity and therefore regulate of litigations in health?	79/123	64.2
P3	Information and evidence	Is the generation of scientific evidence oriented to the identification of health needs of populations an effective measure for the strategical planning of health systems and, therefore, for the regulation of litigations in health?	73/118	61.8
P4	General measures	Is the creation of spaces such as scientific advisory committees to the courts, with the purpose of supporting legal decisions with scientific evidence, an effective measure for decision-making, and also for the regulation of litigation in health?	72/127	56.6
P5	Human resources	Is the timely training of health professionals and judges for decision-making in cases of judicial tutelage an effective strategy to regulate litigation in health?	66/118	55.9
P6	General measures	Is the creation of a public policy that clarifies explicitly the technical and administrative management of the procedures, inputs, interventions, or benefits not included in the set of benefits of the health system, an effective measure to prevent litigation in health?	70/127	55.1
P7	Governance	Can the continuous updating of the services and/or set of features based on the needs and priorities of public health regulate litigations in health?	69/127	54.3
P8	General measures	Is the creation of a strategy that clarifies explicitly which medical inputs and procedures are excluded of the plan of attention in health, as well as the reason of exclusion, an effective measure for the reduction of litigation in health?	67/127	52.7
P9	Information	Can the development and implementation of clinical practice guidelines or protocols be a strategy to regulate litigations in health?	61/118	51.6
P10	Provision of services	Is optimizing the waiting times for the provision of health services an effective measure to reduce the number of litigations in health?	61/122	50.0

### Analysis of the Prioritized Strategies according to Type of Occupation

We identified the trends in response of different individuals, according to their occupation, in 10 prioritized strategies. We found a homogeneous tendency of prioritization of questions of general measures by all actors. In the strategy related to the need of generating scientific evidence oriented to the identification of the needs of the populations, as an effective and efficient strategy for the regulation of litigation in health, health managers had a rate of prioritization significantly greater (18/31 = 58.1%) that the other actors. In the strategy that addressed the optimization of waiting times for the provision of health services as strategy to regulate litigation, active health professionals (15/24 = 62.5%) and decision-makers of the judiciary power (5/11 = 45.5%) had higher rates of prioritization.

The decision-makers of the executive and judiciary powers prioritized the strategies of governance, in which strategies of inclusion of the civil society in the decisions in health stood out, as well as the continuous evaluation of the services offered to the users of the health system.

### Analysis of the Prioritized Strategies according to Country

In general, we observed a tendency to prioritize the strategies referred to in the general measures, governance, and information and evidence measures among countries. Brazil (13/17 = 76.5%) and Colombia (17/33 = 51.5%) prioritized strategies of creating spaces of discussion for decision making; Chile, Costa Rica, Mexico, and Peru prioritized homogeneously: strategies that address the creation of spaces for discussion among the actors in the health system; granting of information on the right to health and the duties of the State in relation to guaranteeing the right to health; generation of scientific evidence for decision-making according to the needs of the populations; and creation of scientific technical advisory committees to the courts. The participants from Argentina prioritized the processes of governance, in terms of accountability (11/21 = 52.4%) and the creation of a regulatory and evaluation process of the interventions and health services that not are included in the basic care plan (11/21 = 52.4%). Those of Uruguay, prioritized strategies seeking to clarify explicitly what interventions and health services are excluded from the basic plan and the reasons for this exclusion (18/28 = 64.3%), followed by the generation of oriented scientific evidence to identify the health needs of populations (15/28 = 53.6%) and the creation of scientific technical advisory committees to support legal decision-making based on scientific evidence (15/28 = 53.6%).

## DISCUSSION

This study is one of the first systematic approaches to identify and prioritize multidisciplinary intervention strategies and different levels of action of health systems to address the judicialization of health. The strategies identified in the published scientific literature were prioritized by a heterogeneous group coming from different countries and contexts. The strategies with greater prioritization scores were those that sought to generate discussion spaces among the different stakeholders, including patients, health professionals and populations in vulnerable situations; also those that ensured the empowerment of the society in relation to the right to health. A second group of strategies highly prioritized is those that establish rules, standards and processes based in scientific data (e.g., the continuous update of the set of continuous benefits, elaboration of guides and protocols of practice, evaluation of technology, among others). A third group of strategies focused on the need to inform and train society, in general, as well as judges and health professional, in particular. Finally, strategies that sought to strengthen health systems to optimize waiting times for the provision of health services, or to improve primary health care, among many others, were considered highly relevant.

In some cases, the participants prioritized intervention strategies that had already been incorporated in some countries in the region as part of the reform processes carried out, such as Colombia, with the regulation of the health market, the costs of provision, and the standardization and updating of the health services plan, which includes updated interventions of the services offered in this country^7^ ^,^ ^13^. Also Argentina, Brazil and Costa Rica, with the creation of spaces of training based in scientific evidence for judges and courts.

The exercise of judicialization (of accountability) highlighted problems in the health system, either in the provision of health services, financing, or rectory or availability of human and structural resources of the system. At the same time, we observed limitations in the judiciary sector to properly perform this role^20^. An analysis point is to consider whether other forms of accountability have been underutilized in the region, benefiting judicial accountability.

In this study, we identified more strategies for the sub-functions of 1) “governance and leadership”, 2) “information”, and 3) “provision of health services”. Probably, the first and third sub-functions are those that have been most explored in relation to the causes of litigation in health in the contexts where they have been studied^29^ ^,^ ^54^. Others authors have asserted that the financing of the health system is the angular stone for a proper provision of health services^8^. However, this had poor prioritization on the part of the participants of this study.

The judicialization of health is a social response to a need that is not met by the health system. Litigations occur frequently as a result of the lack of health coverage, of limitations on access or inequality of access or use, or use, and also detriment in the quality of the care^50^ ^,^ ^d^. In that sense, accomplishing universal health coverage would strengthen the processes of provision of health services, and increase not only the population coverage but the access and the quality of such services by means of a sustainable financial investment, reducing thus the possibility of incurring in expenditure from the budget and, accordingly, in catastrophic expenditure^q^.

There were statistically significant differences of some strategies regarding the function of the respondents. For example, the managers could understand better the role of evidence in decision-making and, thus, of prioritization. We also observed differences in the strategy “optimization of waiting times”, by providers of the health and justice sectors, since the vision of the latter was related to the direct contact between professionals and patients and with the receiving of judiciary demands due to this contact. To ensure timely and proper availability of services, regulate the introduction of new technologies and guarantee equality in access, financing and adequate spending in health are also considerations raised by other authors^r^.

The integrality of the strategies for any action in the health system is necessary to ensure their effectiveness. For example, to think of an intervention on the role of governance and leadership will have impact and adjustments in other functions, both for the provision of services, such as human resources and financing. In the region, an example of this is the implementation of the Integrated Networks of Health Services (RISS), proposed to reduce the excessive fragmentation of health services, thus reducing the difficulties in access and low quality in the provision of services, increasing the efficiency of the system^28^ ^,^ ^s^.

Some strategies identified in this study present relevant scientific evidence that could support its implementation in the countries of the region, taking into account this context^40^ ^,^ ^46^.

### Limitations of the Study

Although we sought to identify participants from various sources, it is not possible to assure the representativeness of the findings. It is difficult to establish the total number of people who had access to the survey on the platform. We estimate that about 35.0% of those who had access to the survey answered it. On the other hand, the low rate of response of participants from Brazil could be due to a language barrier, since the data collection instrument was translated from Spanish into English, but not to Portuguese. Likewise, the representativeness among countries was differential, which could generate a possible selection bias. However, countries that had more representatives were those that, in the literature review, presented more processes of litigation and scientific evidence with regarding judicialization of health. Even having made a systematic search of articles which proposed strategies, it is likely that various strategies have not been identified, and therefore, evaluated by the participants. The survey had a free field so that participants could provide additional strategies, but this resource was little used by them.

## CONCLUSIONS

It is possible to identify priority strategies to effectively guarantee the right to health by the proper performance of health systems. While Table 4 lists the 10 prioritized strategies as most important by respondents in general, we should seek to develop comprehensive strategies contextualized according to the type of model of system of each country. Strategies that are implemented to move toward universal coverage in health can be effective for the reduction of litigations in health in the region, since the dimensions of universal coverage deal with the provision of health services and the financing of such provision.

## Footnotes

a Naciones Unidas, Asamblea General. El derecho de toda persona al disfrute del más alto nivel posible de salud física y mental: informe provisional del relator. New York; 2006. (A/61/338).

b World Health Organization. Social Health Insurance: sustainable health financing, universal coverage and social health insurance: report by the Secretariat: Fifty-eight World Health Assembly. Provisional agenda item 13.16. New York; 2005. (A58/20). Available from: http://apps.who.int/iris/bitstream/10665/20302/1/A58_20-en.pdf

c Iunes R, Cubillos-Turriago L, Escobar ML. Universal health coverage and litigation in Latin America. Washington (DC): World Bank; 2012 [cited 2015 Oct 25]. Available from: http://siteresources.worldbank.org/INTLAC/Resources/257803‐ 1269390034020/EnBreve_178_English_Web.pdf

d Organización Mundial de la Salud. Informe sobre la salud en el mundo: la financiación de los sistemas de salud: el camino hacia la cobertura universal. Ginebra; 2010 [cited 2012 Apr]. Available from: http://whqlibdoc.who.int/whr/2010/9789243564029_spa.pdf

e Savigny D, Taghreed Adam, editors. Systems thinking for health systems strengthening. Geneva: Alliance for Health Policy and Systems Research, WHO; 2009 [cited 2014 Jan]. Available from: http://apps.who.int/iris/bitstream/10665/44204/1/ 9789241563895_eng.pdf?ua=1

f Instituto del Banco Mundial. Iniciativa regional sobre priorización, equidad y mandatos constitucionales: una plataforma sostenible para fomentar coaliciones que impulsen la realización progresiva del derecho a la salud. Washington (DC); 2011 [cited 2012 Apr]. Available from: http://wbi.worldbank.org/wbi/Data/wbi/wbicms/files/drupal-acquia/wbi/spanish_progress_derecho_noreg.pdf

g Acosta OL. La judicialización del derecho a la salud en Colombia. In: Comisión Económica para América Latina; Instituto Centroamericano de Estudios Fiscales; Analytical Spectral Devices; Agencia Española de Cooperación Internacional para el Desarrollo, editores. Panel IV: Perspectivas de los Sistemas de Salud en Seminario Internacional Repensar lo Social en Tiempos de Crisis; 2009 mayo 28-29; Antigua, Guatemala. CEPAL; 2009 [cited 2013 Aug]. Available from: http://www.eclac.cl/dds/noticias/paginas/2/36122/OlgaLuciaAcosta.pdf

h Colombia, Defensoría del Pueblo. La tutela y el derecho a la salud: causas de las tutelas en salud 1999-2003. Bogotá; ano [cited 2014 Jan]. Available from: http://www.defensoria.gov.co/attachment/142/Causas de las tutelas en salud.pdf

i Colombia, Defensoría del Pueblo. La tutela y el derecho a la salud, período 2003-2005. Bogotá; 2007. Available from: http://www.defensoria.gov.co/es/nube/noticias/291/Informe-defensorial-sobre-la-Tutela-y-el-Derecho-a-la-Salud-Informe-defensorial-Salud-Derechos-Humanos.htm

j Rossi F. El precio exagerado de los medicamentos. *Razón Pública*, 27 Sep 2010 [cited 2010 Dec 20]. Available from: http://www.razonpublica.com/index.php/economia-y-sociedad/1408-el-precio-exagerado-de-los-medicamentos

k Procuraduría General de la Nación y DeJusticia, op. cit.; López Medina D. ‘Sistema de salud’ y ‘derecho a la salud’: historia de su interrelación en la jurisprudencia constitucional. Bogotá: Universidad de los Andes, 2008 [cited 5 Oct 2009]. p.42-8. Available from: http://derecho.uniandes.edu.co/derecho_uniandes/ export/derecho_uniandes/facultad/rel_eventos3.htmll

l Colombia, Defensoría del Pueblo. La tutela y el derecho a la salud y a la seguridad social 2014. Bogotá: Defensoria del Pueblo; 2015 [cited 2012 Apr]. Available from: http://www.defensoria.gov.co/public/pdf/LatutelaylosderechosalaSalud.pdf

m Romero LC. Judicialização das políticas de assistência farmacêutica: o caso do Distrito Federal. Brasília (DF): Consultoria Legislativa do Senado Federal; 2008 [cited 2012 Apr]. (Textos para discussão, 41). Available from: http://www12.senado.gov.br/publicacoes/estudos-legislativos/tipos-de-estudos/textos-para-discussao/td-41-judicializacao-das-politicas-de-assistencia-farmaceutica-o-caso-do-distrito-federal

n Perlingeiro R. La tutela judicial del derecho público a la salud en Brasil. el.Dial.com Biblioteca Juridica. 2013 [cited 2014 Jan];16(3722). Available from: http://papers.ssrn.com/sol3/papers.cfm?abstract_id=2239840

o Pepe VLE, Ventura M, organizadores. Manual de indicadores de avaliação e monitoramento das demandas judiciais de medicamentos. Rio de Janeiro: Escola Nacional de Saúde Pública da Fundação Oswaldo Cruz; 2011 [cited 2014 jan]. Available from: http://www5.ensp.fiocruz.br/biblioteca/dados/txt_975659982.pdf

p Vargas Lorenzo I. Barreras en el acceso a la atención en salud en modelos de competencia gestionada: un estudio de caso en Colombia [these]. Bellaterra: Facultat de Medicina de la Universitat Autònoma de Barcelona; 2009.

q World Health Organization. World health report 2000: health systems: improving performance. Geneva; 2000. Available from: http://www.who.int/whr/2000/en/whr00_en.pdf?ua=1

r República de Colombia, Ministerio de la Protección Social. Política farmacéutica nacional. Bogotá: OPAS; 2012 [cited 2012 May]. Available from: https://www.minsalud.gov.co/Documentos%20y%20Publicaciones/Pol%C3%ADtica%20Farmacéutica%20Nacional.pdf

s World Health Organization. Everybody’s business: strengthening health systems to improve health outcomes: WHO’s framework for action. Geneva; 2007. Available from: http://www.who.int/healthsystems/strategy/everybodys_business.pdf
